# Effects of different fresh gas flows on carboxyhemoglobin levels: non-invasive carbon monoxide monitoring: A randomized clinical trial

**DOI:** 10.15537/smj.2022.43.8.20220424

**Published:** 2022-08

**Authors:** Bengü G. Köksal, Gamze Küçükosman, Pişkin Özcan, Çağdaş Baytar, Keziban Bollucuoğlu, Rahşan D. Okyay, Hilal Ayoğlu

**Affiliations:** *From the Department of Anesthesiology and Reanimation, Faculty of Medicine, Zonguldak Bülent Ecevit University, Zonguldak, Turkey.*

**Keywords:** anesthesia, carbon monoxide, carboxyhemoglobin, cigarette smoking

## Abstract

**Objectives:**

To observe the effect of different fresh gas flows (FGF) on carboxyhemoglobin (COHb) levels non-invasively and continuously and to determine the contribution of the smoking status to intraoperative carbon monoxide (CO) accumulation and respiratory complications.

**Methods:**

A total of 64 patients were included in the study. Carboxyhemoglobin level was monitored non-invasively from the fingertip. Patients were divided into 2 according to the FGF as low-flow anesthesia (LFA; Group L) and high flow anesthesia (Group H). Each group was divided again into 2 groups as smokers and non-smokers. Carboxyhemoglobin and and the respiratory complications that occurred in the post-anesthesia care unit were recorded.

**Results:**

The mean COHb values were significantly higher in Group L between 30th and 210th minutes. Furthermore, in Group L, intraoperative COHb levels were significantly higher in smokers compared to non-smokers in all periods. In group H, no difference was observed between smokers and non-smokers in terms of COHb levels after 60 minutes and also preoperative COHb levels of the patients developed respiratory complication was higher.

**Conclusion:**

If the CO_2_ absorbent is properly preserved in patients who are administered LFA, there will be no risk of CO accumulation even in chronic smokers.

**ClinicalTrials.gov REG. No.: NCT04832256**

**T**he term “low-flow anesthesia” (LFA) is used to define inhalation anesthesia techniques with a minimum rebreathing rate of 50%. It occurs when the fresh gas flow (FGF) rate is reduced below 2 L/minute using modern rebreathing systems.^1^

The main advantages of low-flow techniques are reduction in cost, better conditioning of the inspired anesthetic gases in terms of temperature and humidity, and low atmospheric pollution. In addition, they are known to have undesirable effects such as increased absorbent consumption, lack of knowledge on the exact anesthetic gas composition in the ventilation system, and unwanted gas accumulation in the respiratory system (carbon monoxide [CO] and methane).^2^ Serious CO poisoning (carboxyhemoglobin [COHb] 36%) has been reported on account of drying of the carbon dioxide (CO_2_) absorbent in intermediate flow anesthesia wherein a 2 L/minute FGF is applied with desflurane.^3^ Carbon monoxide is a tasteless, odorless, and colorless gas with biological effects on various tissues and organs. Sources of CO include insufficiently ventilated heat sources, car exhausts, faulty furnaces, methylene chloride exposure, cigarette smoke, and smoke from fires.^4^

Carbon monoxide causes hypoxia by generating COHb and shifting the oxyhemoglobin dissociation curve to the left. The affinity of CO for hemoglobin is 200 times that of oxygen (O_2_), which causes the generation of COHb with CO inhaled in relatively low amounts. Exposure to CO also causes inflammation, which results in neurological and cardiac damage.^5^

A COHb level above 3% in non-smokers or above 10% in smokers confirms exposure to CO. Carbon monoxide levels of ≥3% may unfavorably affect high-risk groups such as elderly people, pregnant women, fetuses, babies, or patients with cardiovascular or respiratory tract disorders.^6,7^

The measurement of arterial blood gases in cases of CO poisoning provides information on gas exchange, metabolic acidosis, and COHb sufficiency. However, measurement of venous COHb levels is sufficient for diagnosis.^8^

Pulse CO-oximetry is a continuous and non-invasive method used to measure the levels of various blood components, including COHb. Measurements are usually carried out by placing a sensor on the fingertip.^9^

In this study, our primary aim was to observe the effect of different FGF (1 and 4 L/minute) on COHb levels by non-invasive and continuous measurements in anesthesia applications; and to determine the contribution of smoking status to intraoperative CO accumulation and the respiratory complications that may occur during the recovery period.

## Methods

This prospective randomized study was carried out at Zonguldak Bülent Ecevit University Health Training and Research Center, Turkey from January 2016 to December 2018. In total, 64 patients from the American Society of Anesthesiologists (ASA) I-II risk group with an age range of 18-65 years who underwent 2-4 hours of elective nasal surgery were appropriate for inclusion in the study. Patients with cardiovascular disease, diabetes mellitus, renal or hepatic insufficiency, chronic obstructive pulmonary disease, opioid sensitivity, personal or family history of malignant hyperthermia or delayed emergence, morbid obesity, alcohol addiction, pregnancy and lactation, and allergy to the study drugs were excluded from the study. Approval from the local clinical research ethics committee (2015-67-07/07) was obtained. Written informed consents were received from all patients.

We defined smokers as those who had smoked at least one cigarette per day in the past month. The patients were not allowed to smoke on the morning of the surgery; however, patients who did not smoke in the last 12 hours were not included in the study because COHb levels were associated with the time elapsed after smoking the last cigarette based on the half-life of COHb.

Anesthesiologists were blinded to the study design when they carried out LFA or high-flow anesthesia (HFA). No premedication was administered to any of the patients. In addition to routine ASA monitoring, the bispectral index and COHb level were monitored non-invasively using the Masimo Radical-7 Pulse CO-Oximeter (Masimo Corporation, ABD) from the fingertip. Pulse oximeter (SpCO) readings detected COHb. The canister of CO_2_ absorbent (Soda lime, Drägersorb^®^ 800) was replaced before each procedure. Every patient was closely observed for inspired and expired oxygen, inhalation agents, and minimum alveolar concentrations.

In all patients, general anesthesia induction was initiated after preoxygenation at 10 L/minute via a mask for 5 minutes with 100% oxygen. After anesthesia induction, endotracheal intubation was carried out accordingly for all patients. An anesthesiologist, who was not part of the team involved in the study, made a random allocation sequence using numbered, opaque, sealed envelopes. These envelopes were opened by another anesthetist who was not included in the study, anesthesia was maintained, and participant data were thus recorded. After intubation, the patients were separated into 2 groups: LFA group and HFA group. Again, the patients who were administered HFA (Group H, n=32) were divided into 2 groups according to whether they smoked or not: smoking group (Group I, n=12) and non-smoking group (Group II, n=20). Patients who were administered LFA (Group L, n=32) were also divided into 2 groups: smoking group (Group III, n=9) and non-smoking group (Group IV, n=23). During the initial 10 minutes, desflurane was administered at a concentration of 6% in 50% O_2_/air mixtures at a rate of 4 L/minute in Group H, and Group L and remifentanil infusion was started at a rate of 1 µg/kg/minute. Group L was switched to a low flow (1 L/minute) after 10 minutes. End-tidal CO_2_ (EtCO_2_) was maintained between 30-35 mmHg, and remifentanil infusion was fixed at a rate of 0.1 µg/kg/minute. Desflurane was started at a volume of 6%, and the bispectral index was set at 40-60. The expiration valve was opened, and the FGF rate was increased to 6/L/minute at 10 minutes before the end of the operation. Anesthetic agent inhalation was turned off during suturing and ventilation was carried out manually using 100% O_2_. Neostigmine (0.04 mg/kg) and atropine (0.02 mg/kg) were applied to reverse neuromuscular blockade. Patients with sufficient spontaneous respiration and muscle strength were extubated accordingly.

End-tidal CO_2_, peripheral oxygen saturation (SPO2), COHb, and hemodynamic data of all patients were measured preoperatively (T0), after intubation (T1), and at 10 (T2), 20 (T3), 30 (T4), 45 (T5), 60 (T6), 90 (T7), 120 (T8), 150 (T9), 180 (T10), and 210 (T11) minutes.

Respiratory complications occurring in the post-anesthesia care unit (PACU) included arterial desaturation, coughing, laryngospasm, bronchospasm, and apnea/breathholding.

### Statistical analysis

The sample size required for the study was calculated using Power Analysis and Sample Size 11 before initiation of the study. Considering a 95% confidence interval (1-α), 80% test power (1-β), and an effect size of d=0.632. A total of 64 participants were included in the study, with a minimum of 32 participants in each group.^10^

Statistical Package for the Social Sciences, version 22.0 (IBM Corp., Armonk, NY, USA) was used to analyze the data obtained in this study. Conformity of the normal distribution was evaluated using the Shapiro-Wilk test. Descriptive statistics are presented as means or medians for continuous variables according to the normality distribution. Categorical variables are expressed as percentages and counts. The Chi-square test was carried out to compare qualitative data such as gender and ASA score. When hemodynamic parameters, body mass index, duration of anesthesia and surgery, and amounts of remifentanil and desflurane consumed were compared between the 2 groups, normally distributed variables in independent groups were analyzed using the t-test and non-normally distributed variables were analyzed using the Mann-Whitney-U test. The generalized linear model method was carried out to examine the effect of the main effects and interactions of the group, smoking, and time on COHb values, and the Bonferroni test was carried out for multiple comparisons. Statistical significance was set at a *p*-value of <0.05.

## Results

**Figure 1:**
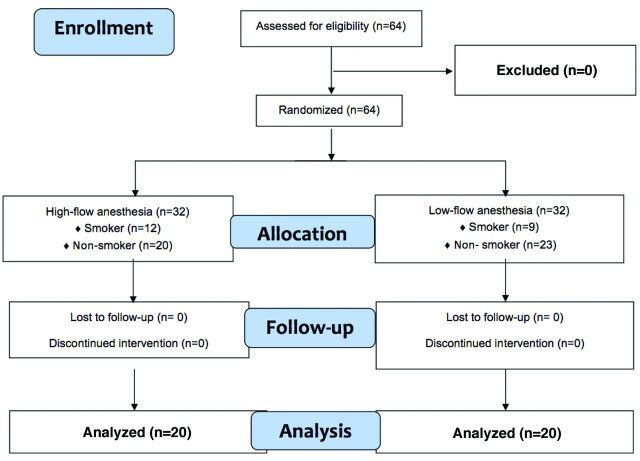
- Flow diagram of the study (CONSORT).

In total, 64 patients, 35 men and 29 women with ages ranging from 18-67 years were included in this study. A flow diagram of the allocation, follow-up, and patient analysis are shown in Figure 1. The mean age was 40.64±11.36. While no significant difference was detected between the groups in terms of demographic characteristics, amount of cigarettes smoked, duration of anesthesia, operation time, ASA score, and total amount of remifentanil consumed. A significant difference was found in the total amount of desflurane consumed by these groups (*p*<0.001; Table 1).

**Table 1:** - Sociodemographic variables and intraoperative data.

Variables	Group H (n=32)	Group L (n=32)	*P*-values
Age (year)	41.90±9.82	39.37±12.75	0.377
BMI (kg/m2)	25.65±3.36	25.54±3.73	0.895
Gender, female/male (n)	14/18	15/17	0.802
ASA score I/II (n)	15/17	13/19	0.614
Amount of cigarettes, pack/year (median)	0.5 (0-45)	2.5 (0-35)	0.600
Duration of surgery (minute)	166.73±32.17	184.13±41.47	0.075
Duration of anesthesia (minute)	175.16±32.50	192.90±42.94	0.077
Total remifentanil consumption (µg)	1242.73±319.56	1377.80±392.94	0.150
Total desflurane consumption (ml)	263.43±135.09	87.73±40.96	<0.001

Values are presented as mean ± standard deviation (SD). BMI: body mass index, ASA: American Society of Anesthesiologists, µg: microgram, n: number of patient, Group L: low-flow anesthesia, Group H: high-flow anesthesia

A comparison of COHb values by group, smoking status, and time is summarized in Table 2. The group main effect was found to be statistically significant for COHb values (*p*<0.001). While the mean COHb level in group H was 0.759, that in group L was 1.365. The mean COHb values differed between the groups. Group and time interactions were found to be statistically significant for the COHb values (*p*=0.008). While the highest average value was 1.584 in Group L at the 210th minute, the lowest average value was 0.541 in Group H at the 210th minute.

**Table 2:** - Comparison of carboxyhemoglobin values by group, smoking status, and time.

Variables	F	DF	*P*-values
Group	84,132	1	<0.001
Smoking status	191,568	1	<0.001
Time	11,707	11	0.386
Group - smoking status	7,002	1	0.008
Group - time	25,541	11	0.008
Smoking status - time	37,12	11	<0.001
Group - smoking status - time	2,09	11	0.998

DF: degrees of freedom, F: test statistic

The mean COHb values were significantly higher in Group L than in Group H between the 30th and 210th minutes (Table 3). When the COHb values were examined according to time in Group H, a significant difference was found between the COHb values measured at T0 and all measurement times except T1, T2, and T3; between the COHb values measured at T1, T5, T9, T10, and T11; and between the COHb values measured at T2, T9, T10, and T11 (*p*<0.05). However, there was no significant difference between any of the measurement times in Group L (Table 3).

**Table 3:** - Comparison of the mean carboxyhemoglobin values of the groups by time.

Time	Group H	Group L	*P*-values^*^
T0 COHb	1.36±1.14	1.40±1.23	0.874
T1 COHb	1.11±1.06	1.12±1.32	0.983
T2 COHb	0.99±1.00	1.25±1.34	0.385
T3 COHb	0.85±0.93	1.36±1.35	0.085
T4 COHb	0.72±0.88	1.27±1.23	0.046
T5 COHb	0.60±0.76	1.26±1.10	0.007
T6 COHb	0.61±0.74	1.39±1.21	0.003
T7 COHb	0.65±0.73	1.30±1.05	0.006
T8 COHb	0.59±0.65	1.44±1.17	0.001
T9 COHb	0.51±0.63	1.45±1.24	<0.001
T10 COHb	0.54±0.62	1.51±1.31	<0.001
T11 COHb	0.54±0.61	1.58±1.38	<0.001
*P*-values^**^	<0.05	>0.05	

Values are presented as mean ± standard deviation (SD).

^*^ Inter-group comparison,

^**^ intragroup comparison, Group H: high-flow group, Group L: low-flow group, COHb: carboxyhemoglobin

Patients undergoing LFA and HFA were divided into subgroups of smokers and non-smokers. The preoperative mean COHb level was 2.20 in smokers and 0.5 in non-smokers.

The group-smoking status interaction was shown to have a statistically significant effect on COHb values (*p*=0.008). In Group H, the mean COHb value of smokers was 1.129 and 0.390 of non-smokers. In Group L, the mean COHb value of smokers was 1.908 and 0.818 of non-smokers.

In Group L, the intraoperative COHb values were significantly higher in smokers than in non-smokers at all periods. In Group H, COHb levels were higher from the preoperative period to the intraoperative 60th minute in smokers. The mean COHb levels of the subgroups over time are shown in Table 4.

**Table 4:** - Comparison of carboxyhemoglobin values of groups according to smoking status.

Time	Group H			Group L		
	Group I (n=12)	Group II (n=20)	*P*-values	Group III (n=9)	Group IV (n=23)	*P*-values
T0 COHb	2.23±0.88	0.49±0.41	<0.001	2.10±1.18	0.71±0.85	0.001
T1 COHb	1.80±1.08	0.43±0.41	<0.001	1.85±1.28	0.39±0.90	0.001
T2 COHb	1.50±1.15	0,47±0.40	0.003	1.85±1.24	0,64±1.19	0.009
T3 COHb	1.26±1.12	0.43±0.40	0.012	1.95±1.27	0.76±1.18	0.011
T4 COHb	1.19±0.98	0.26±0.42	0.002	1.78±1.15	0.76±1.13	0.018
T5 COHb	0.98±0.84	0.21±0.41	0.004	1.70±1.07	0.83±0.98	0.024
T6 COHb	0.91±0.87	0.31±0.44	0.022	1.79±1.05	0.87±1.14	0.025
T7 COHb	0.85±0.85	0.45±0.53	0.089	1.68±0.99	0.91±1.00	0.036
T8 COHb	0.72±0.74	0.45±0.53	0.252	1.81±1.06	1.06±1.18	0.027
T9 COHb	0.64±0.72	0.38±0.50	0.305	1.94±0.94	0.92±1.14	0.034
T10 COHb	0.70±0.70	0.38±0.50	0.557	2.10±1.42	0.92±1.18	0.010
T11 COHb	0.70±0.68	0.37±0.50	0.649	2.13±1.35	1.50±1.76	0.032
P-values^*^	<0.05	>0.05		>0.05	>0.05	

Values are presented as mean ± standard deviation (SD).

* Intragroup comparison, COHb: carboxyhemoglobin

The incidence of respiratory complications was 28.1% in Group H and 9.4% in Group L. There was no significant difference between the groups with regard to respiratory complications (*p*>0.05; Table 5). Among the patients who developed respiratory complications, one patient in Group L and 6 patients in Group H had a history of smoking. The difference between the mean preoperative COHb levels of the patients who developed (2.09±1.20) and those who did not develop (1.22±1.10) respiratory complication were found to be statistically significant (*p*=0.019). However, no differences were observed at other time intervals.

**Table 5:** - Incidence of respiratory complications in the postanesthesia care unit: high-flow versus low-flow anesthesia.

Variables	Group H (n=32)	Group L (n=32)	*P*-values
*Complications*			
No	23 (71.9)	29 (90.6)	0.055
Yes	9 (28.1)	3 (9.4)	
Desaturation	1 (3.1)	0 (0.0)	0.055
(SpO_2_<92%)			
Apnea/breath-holding	1 (3.1)	1 (3.1)	
Coughing	6 (18.8)	2 (6.2)	0.257
Bronchospasm	1 (3.1)	0 (0.0)	
Laryngospasm	0 (0.0)	0 (0.0)	-

Values are presented as a number and precentage (%). SpO_2_: peripheral capillary oxygen saturation, Group L: low-flow group, Group H: high-flow group

## Discussion

In our study, although COHb levels were significantly higher in LFA than in HFA, it was observed that these levels were within normal limits and they could be non-invasively measured with the pulse CO-oximeter. Furthermore, in Group L, intraoperative COHb levels were considerably higher in smokers than in non-smokers at all time points. In Group H, COHb levels were higher from the preoperative period to the intraoperative 60th minute in smokers. Our other finding was that the mean preoperative COHb level of patients who developed respiratory complications was higher.

Since CO may also be generated endogenously in the human body as a result of the catabolism of hemoglobin, COHb can be detected in human blood at a rate of 0-5%.^11^ Carbon monoxide poisoning causes a decrease in the oxygen-carrying capacity of hemoglobin, impairment of oxygen distribution, and tissue hypoxia.^12^

With regard to general anesthesia applications, in the oldest case report of CO poisoning, it was stated that the COHb levels measured in arterial blood gas 25 minutes and one hour after the induction of anesthesia in a non-smoking patient were 9.1% and 28%.^13^ Cases of anesthesia-dependent CO exposure continued to be reported afterwards. In some cases, it was reported that the CO concentrations in the breathing circuit exceeded 1000 ppm and that the COHb levels of the patients reached 30% or more. This was attributed to the drying of the CO_2_ absorbent due to the high gas flow used, since the amount of CO produced is inversely proportional to the water content of the absorbent.^14,15^ In our study, it was found that the mean COHb levels were within normal limits (0-5%) during the operation, including for the smokers. We believe that this was because we used a fresh CO_2_ absorbent that did not contain potassium and sodium hydroxides to prevent the contribution of exogenous CO.

Low-flow anesthesia allows for re-inhalation and preservation of volatile anesthetics. Since the exhaled CO is not excreted or removed from the breathing circuit, patients can inhale the exhaled CO during LFA. Carbon monoxide exposure is inversely proportional to FGF rates; therefore, higher CO levels result from lower FGF levels.^16^

In experimental studies, the effects of different FGF levels on the CO level were examined, and different results were obtained accordingly. While, in a study, it was shown that FGF with a rate <1 L/minute cause higher CO levels, in another study in which 1 L/minute and 4 L/minute FGF were used, it was reported that high FGF (4 L/minute) caused the CO_2_ absorbent to dry out and increase CO production.^16,17^

In the clinical study of Baum et al^18^, while the preoperative COHb concentration was 2.1±1.05 in the minimal flow anesthesia with desflurane, 45 minutes after the flow was reduced from 4.4 L/minute to 0.5 L/minute, the COHb concentration measured to 1.42±1.01 and to 1.41±0.78 after 105 minutes.

In their studies comparing HFA and LFA applications, Avcı et al^19^ found that there was no difference between the COHb levels measured in arterial blood gas at 30 and 90 minutes.

In children, it has been demonstrated that when the FGF is adjusted below the minute ventilation, CO increases to 20 ppm with the simultaneous increase in COHb.^20^ In a study on pediatric patients evaluating the artery blood gas results and the COHb together in LFA (1 L/minute) and HFA (6 L/minute) applications, COHb levels were found to be higher in patients who were applied LFA at the end of ventilation and at the 10th minute of ventilation than in the HFA group, but still within the normal limits.^10^ Nasr et al^20^ aimed to measure the endogenous CO amount inhaled during LFA in infants and children and to determine the factors that affect the CO level in the anesthesia circuit. They connected an electrochemical CO sensor to 2 sampling ports in a breathing circuit to measure the CO level. They showed that, during HFA, exhaled CO levels remained constant, whereas inhaled CO decreased significantly. It was observed that the change in exhaled and inhaled CO was closely related to FGF and that the COHb level significantly increased at the 60th minute of LFA in 2-year-old children.

In the present study, the preoperative COHb concentration at LFA with desflurane was found to be 1.40±1.23, while it was measured as 1.39±1.21 at 50 minutes after FGF was reduced to one L/minute and as 1.44±1.17 110 minutes after the FGF was reduced to one L/minute, followed by an increase (1.58±1.38) until the 200th minute. In the LFA group, COHb levels were found to be significantly higher than in the HFA group between the intraoperative 30th minute and 210th minute but within the normal limits. The reason for this difference might be because heavy smokers were included in our study. When evaluated within the groups, a decrease in the COHb level was detected over time in the HFA group. We believe that the reason for the decrease in COHb level is related to FGF (minute ventilation).

Cigarette smoke is an important source of CO. While the COHb level of regular smokers can vary between 3-8%, it has been reported that this rate may reach up to 10% in heavy smokers, drivers working in heavy traffic, and boiler room workers.^5^ In a study where anesthesia was applied with a closed circuit for 6 hours, the post-anesthesia COHb concentration was found to be 0.5-1.5% in non-smokers, while it reached up to 3% in smokers.^21^

Tang et al^22^ continuously measured CO using the electrochemical method and concluded that chronic smokers, patients who smoked before surgery, and patients with higher body weight are associated with increased CO concentrations and that the increase in gas flow rates may reduce CO concentrations. They demonstrated in their regression model that the highest and mean CO concentrations of smokers were 11.5 ppm and 7.5 ppm higher than of non-smokers (*p*<0.01) and that for every one-minute increase in the gas flow rate, a decrease of 7.7 ppm was observed for the peak CO and 5.9 ppm for the mean CO decrease.

In our study, the preoperative COHb levels in smokers were 2.20. In Group L, intraoperative COHb levels were significantly higher in smokers than in non-smokers at all periods. In Group H, COHb levels were higher from the preoperative period to the intraoperative 60th minute in smokers. After 60 minutes, no difference was observed between smokers and non-smokers in terms of COHb levels. We believe that the reason for the COHb levels being lower than expected despite the inclusion of heavy smokers may be the fact that the number of cigarettes smoked within 12 hours was small and that smoking was not allowed on the operation day.

It has been reported that tracheobronchial physiology and the mucociliary activity was preserved better in LFA than in HFA.^23^ There are also studies showing that the pulmonary function test results were similar in LFA and HFA after surgery.^24^

In our study, although postoperative respiratory complications were less frequent in the LFA group than in the HFA group, the difference was not significant.

Smoking is a risk factor for many perioperative complications, such as cardiovascular and respiratory complications and surgical site infection.^25^

In a study investigating the effects of exhaled CO levels on preoperative smoking status and perioperative respiratory complications in patients who underwent laparoscopic cholecystectomy, Ozgunay et al^26^ have demonstrated that the incidence of bronchospasm during extubation, hypoxia, cough, and apnea in the PACU significantly increased in the smoking group. They reported that the incidence of cough among smokers was 41.3% and 6.5% in non-smokers. A one-unit increase in CO level in the exhaled breath was associated with a 1.16-fold increase in hypoxia.

The most common respiratory complication in our study was cough. Cough was observed in 21.9% of smokers and 6.2% of non-smokers. The difference between the mean preoperative COHb levels of patients who developed respiratory complications and those who did not was also found to be statistically significant.

The most common way to measure CO exposure in hospitals is to use a laboratory CO-oximeter. Blood samples collected from a vessel are injected into the laboratory CO-oximeter. Carboxyhemoglobin level is measured invasively using a spectrophotometric method combined with blood gas analysis.^27^ Standard pulse oximeters cannot distinguish hemoglobin bound to CO from oxyhemoglobin. Therefore, pulse oximeters erroneously overestimate arterial oxygen saturation in the presence of high CO in the blood.^28^ However, easily accessible non-invasive methods have emerged in recent years. Carbon monoxide breath analyzers have been reported to be fast and reliable for determining COHb levels.^29^

The latest technology used for detecting CO poisoning measures blood CO saturation levels by using 7+ wavelengths of light to distinguish various forms of hemoglobin (oxy-, deoxy-, carboxy-, and met-), allowing the detection of CO levels in the bloodstream continuously and non-invasively.^30^ In a study of healthy volunteers, the Radical 7 pulse CO-oximeter was shown to accurately detect hypoxemia with both low and high COHb levels when oxygen saturation is greater than approximately 85%.^31^

In our study, this method was preferred since it does not require a blood sample, is time-saving during laboratory analysis, and continuously shows the CO level. This method also allows data to trend over time.

### Study limitations

We were not able to measure the temperature/humidity of the gas beyond the “Y” piece. Another limitation is that COHb monitoring was not continued after extubation. The method that is considered the gold standard for determining COHb levels has not been considered, and its consistency with the non-invasive method that we applied has not been demonstrated accordingly. Another important limitation was the relatively small number of smokers who participated in our study. Prospective studies with large sample sizes are needed to determine the effect of smoking status on COHb levels in LFA patients.

In conclusion, changes in COHb levels were monitored non-invasively using a pulse CO-oximeter in our study, wherein we administered high- and low-flow desflurane anesthesia to patients. We concluded that if the CO_2_ absorbent is properly preserved in patients who are administered LFA, there will be no risk of CO accumulation, even in chronic smokers. We believe that LFA can be used safely for both ecological and economic reasons, or for patients with low COHb tolerance, provided that CO levels are continuously monitored.
